# The *Rose-comb* Mutation in Chickens Constitutes a Structural Rearrangement Causing Both Altered Comb Morphology and Defective Sperm Motility

**DOI:** 10.1371/journal.pgen.1002775

**Published:** 2012-06-28

**Authors:** Freyja Imsland, Chungang Feng, Henrik Boije, Bertrand Bed'hom, Valérie Fillon, Ben Dorshorst, Carl-Johan Rubin, Ranran Liu, Yu Gao, Xiaorong Gu, Yanqiang Wang, David Gourichon, Michael C. Zody, William Zecchin, Agathe Vieaud, Michèle Tixier-Boichard, Xiaoxiang Hu, Finn Hallböök, Ning Li, Leif Andersson

**Affiliations:** 1 Department of Medical Biochemistry and Microbiology, Uppsala University, Uppsala, Sweden; 2 State Key Laboratory for Agrobiotechnology, China Agricultural University, Beijing, China; 3 Department of Neuroscience, Uppsala University, Uppsala, Sweden; 4 INRA, AgroParisTech, UMR1313 Animal Genetics and Integrative Biology, Jouy-en-Josas, France; 5 INRA, UMR 444, Cellular Genetics Lab, Castanet Tolosan, France; 6 INRA, UE1295 PEAT, Nouzilly, France; 7 Broad Institute of Harvard and MIT, Cambridge, Massachusetts, United States of America; 8 Centre de Sélection de Béchanne, St-Etienne du Bois, France; 9 Department of Animal Breeding and Genetics, Swedish University of Agricultural Sciences, Uppsala, Sweden; The Roslin Institute and The Royal (Dick) School of Veterinary Studies, United Kingdom

## Abstract

Rose-comb, a classical monogenic trait of chickens, is characterized by a drastically altered comb morphology compared to the single-combed wild-type. Here we show that *Rose-comb* is caused by a 7.4 Mb inversion on chromosome 7 and that a second *Rose-comb* allele arose by unequal crossing over between a *Rose-comb* and wild-type chromosome. The comb phenotype is caused by the relocalization of the MNR2 homeodomain protein gene leading to transient ectopic expression of MNR2 during comb development. We also provide a molecular explanation for the first example of epistatic interaction reported by Bateson and Punnett 104 years ago, namely that walnut-comb is caused by the combined effects of the *Rose-comb* and *Pea-comb* alleles. Transient ectopic expression of MNR2 and SOX5 (causing the Pea-comb phenotype) occurs in the same population of mesenchymal cells and with at least partially overlapping expression in individual cells in the comb primordium. *Rose-comb* has pleiotropic effects, as homozygosity in males has been associated with poor sperm motility. We postulate that this is caused by the disruption of the *CCDC108* gene located at one of the inversion breakpoints. CCDC108 is a poorly characterized protein, but it contains a MSP (major sperm protein) domain and is expressed in testis. The study illustrates several characteristic features of the genetic diversity present in domestic animals, including the evolution of alleles by two or more consecutive mutations and the fact that structural changes have contributed to fast phenotypic evolution.

## Author Summary

Comb morphology is a trait that shows considerable variability among domestic chickens. The *Rose-comb* mutation causes a drastically altered shape of the comb, whereas the *Pea-comb* mutation leads to a considerable reduction in the size of the comb. The combined effect of *Rose-comb* and *Pea-comb* is a comb shaped like a walnut, and the phenotype is consequently named walnut-comb. Both Pea-comb and Rose-comb are caused by structural changes in the genome leading to altered expression of important transcription factors. In a previous study we showed that Pea-comb is caused by misexpression of SOX5 during the development of the comb. In this study we report that Rose-comb is caused by a large inversion on chicken chromosome 7. The inversion moves the *MNR2* gene to a new genomic location. This leads to misexpression of MNR2 during comb development, similar to the defect causing Pea-comb. Roosters that are homozygous for the *Rose-comb* inversion show poor sperm motility, and our results suggest that this is caused by the disruption of the CCDC108 gene that is located at one of the inversion breakpoints. CCDC108 is well conserved between chickens and humans, and this study establishes CCDC108 as a candidate gene for sperm motility disorders in humans.

## Introduction

**Figure 1:**
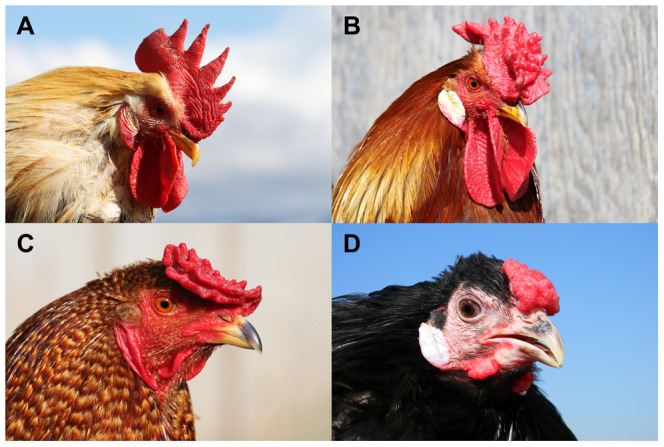
Four comb phenotypes in chickens explained by segregation at the *Rose-comb* and *Pea-comb* loci and their interaction. (*A*) Single-combed wild-type male (*rr pp*), (*B*) Rose-combed male (*R- pp*), (*C*) Pea-combed male (*rr P-*) and (*D*) walnut-combed male (*R- P-*). Photos by Freyja Imsland (A–C) and David Gourichon (D).

Rose-comb (Figure 1) was one of the autosomal dominant traits William Bateson used in his seminal paper describing Mendelian inheritance in animals for the first time [1]. This mutation probably occurred early in the process of chicken domestication, as it is widespread among chicken populations originating in both Asia and Europe, separated for hundreds of years. A few years after the first description of the mode of inheritance of Rose-comb, Bateson and Punnet [2] reported the first case of epistatic interaction between genes as they demonstrated that individuals carrying both the *Rose-comb* and *Pea-comb* alleles exhibit the walnut-comb phenotype (Figure 1). Rose-comb has been described in many breeds and shows extensive phenotypic variability (Figure S1). Most attention has been paid to variation in surface characteristics and texture, angle and number of posterior spikes [3]–[5]. Thus, Rose-comb variability indicates that comb morphogenesis is influenced by several genes and represents an excellent model to study interactions between developmental genes.

Numerous reports have documented reduced male fertility associated with the *Rose-comb* allele [6]–[8]. Gradually it was elucidated that defective sperm motility in roosters homozygous for *Rose-comb* is the cause of the observed poor fertility and duration of fertility [9]–[15]. This reduced motility is thought to result in sperm from a homozygous *Rose-comb* rooster (*RR*) being outcompeted by sperm from roosters carrying the wild-type allele (*Rr* and *rr*) in promiscuous mating or heterospermic insemination experiments [16], [17]. Heterozygous Rose-comb roosters show normal fertility and transmit their *Rose-comb* and *wild-type* alleles to equal number of progeny. Fertility in the hen has been shown to be unaffected by her genotype at the *R* locus [18]. The unaffected fertility of the heterozygous rooster, combined with the effects of sperm competition, has historically confounded breeders' attempts to establish flocks that breed true for Rose-comb, resulting in an equilibrium of allele frequencies that gives rise to about 15% single-combed chicks in each generation [19].

In the present study, we show that Rose-comb is caused by a large structural rearrangement that leads to transient ectopic expression of an important transcription factor in the chicken, the Mnx-class homeodomain protein MNR2 [20]. This resembles our previous discovery that the *Pea-comb* mutation constitutes a copy number expansion in intron 1 of *SOX5* leading to transient ectopic expression of SOX5 [21]. We also postulate that the sperm motility defect observed in males homozygous for *Rose-comb* is due to the same structural rearrangement disrupting the *CCDC108* gene.

## Results

### Linkage mapping reveals that Rose-comb is associated with suppressed recombination

Linkage mapping was performed using a pedigree consisting of two F_0_ males heterozygous for *Rose-comb*, sixteen F_0_ single-combed females, and 383 F_1_ progeny segregating for *Rose-comb* (Rose-comb 50.7%, single-comb 49.3%). Already in 1940 *Rose-comb* was mapped to chicken linkage group I [22] and in a recent study *Rose-comb* was assigned to chicken chromosome 7 (GGA7) [23]. Our linkage data, presented in Table 1, confirmed the assignment to GGA7 and revealed suppression of recombination in *Rose-comb* heterozygotes as no recombination event was detected over a 7 Mb region, from position 16.1 Mb to 23.4 Mb on GGA7, despite the chicken consensus linkage map indicating a distance of 50 cM across this region [24]. This suppression of recombination associated with Rose-comb was confirmed in a second pedigree, a Chinese Silkie x White Plymouth Rock intercross (Text S1).

**Table 1:** Two-point linkage analysis of the *Rose-comb* locus using 11 markers on chicken chromosome 7.

Marker^1^	Rec. fracs.	LOD	galGal3 Marker Location (bp)	Marker type
MCW0120	0.11	27.1	11,903,615–11,903,662	Microsatellite
ADL0107	0.08	33.6	12,980,283–12,980,306	Microsatellite
C7C19.217	0.01	105.3	14,557,641	SNP
C7C19.249	0.00	112.9	16,077,352	SNP
C7C19.253	0.00	113.5	16,434,005	SNP
KIAA1715	0.00	109.9	17,510,907	SNP
PTD004	0.00	110.5	18,268,510	SNP
C7C19.442	0.00	112.9	18,865,430	SNP
C7C19.559	0.00	113.8	21,118,705	SNP
C7C107.36	0.00	112.9	23,443,405	SNP
C7C15.222	0.36	5.8	33,000,168	SNP

1 SNP markers are defined in Table S2.

### Detection and characterization of a large inversion associated with *Rose-comb*

The observed suppression of recombination suggested that *Rose-comb* might be associated with an inversion. This hypothesis was strongly supported by a SNP screen using an Illumina 60K SNP array [25] of *Rose-comb* homozygotes from four Chinese chicken breeds, showing complete homozygosity for all SNPs in the interval 16,424,096 bp to 23,854,241 bp despite this region showing normal levels of heterozygosity in wild-type birds (Figure S2).

**Figure 2:**
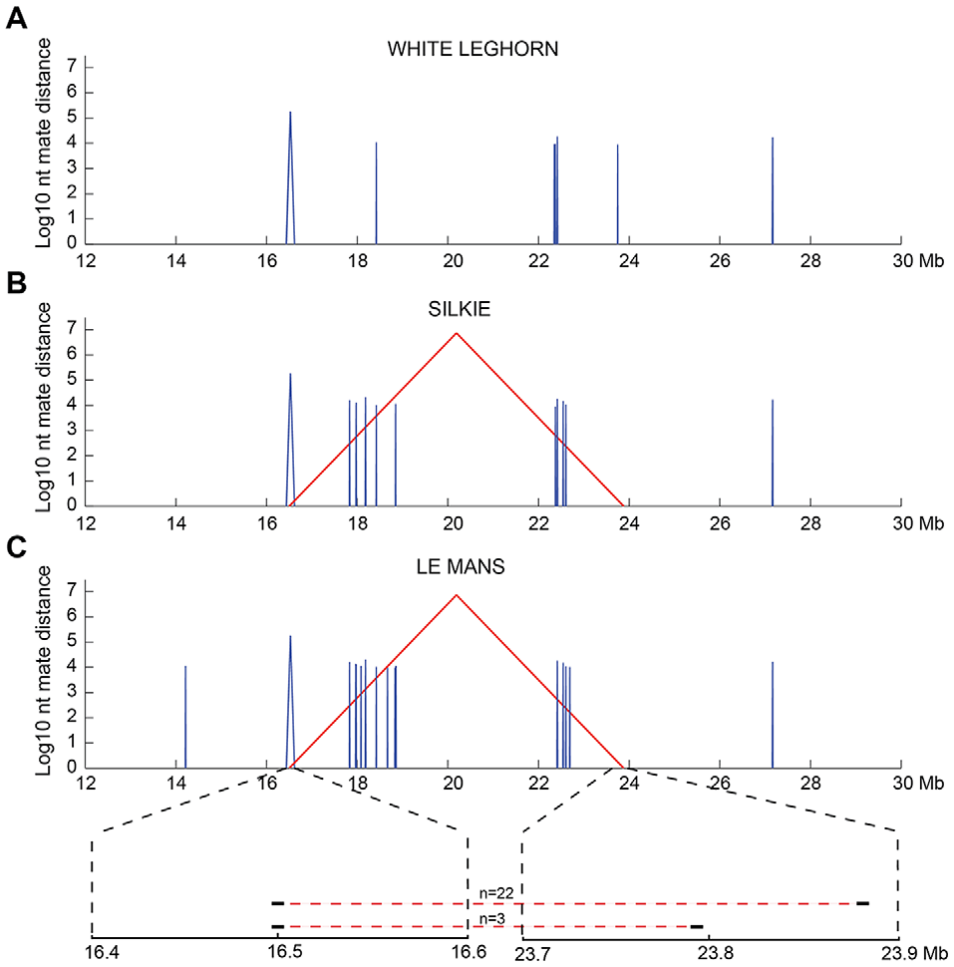
Candidate structural variants identified from whole-genome resequencing. Mate-pair information was used to plot structural variants in the region of interest (chr7:12–30 Mb) for sequenced pools of (A) single-combed White Leghorn, (B) Rose-combed Chinese Silkie (C) Rose-combed Le Mans. Structural variants were defined as 1.5 kb windows where at least 25% of the mate pairs had mapping distances exceeding ten standard deviations above the average insert size and those > = 25% that were mapped within 1 kb of each other. Y-axis indicates the size of candidate structural variants in log_10_ base pairs. X-axis indicates the genomic coordinates of the pairs supporting structural variants. Red colour indicates mates that map to different strands, indicative of inversion. Blue colour indicates mates that map to the same strand, indicative of a deletion or duplication. The structural variants uniquely observed in the two Rose-combed pools included an inversion candidate, stretching between approximately 16.50–23.88 Mb. In the Le Mans pool an additional inversion candidate was also observed between 16.50–23.79 Mb, supported by three read pairs. This is depicted at the magnified region at bottom of (C). Candidate structural changes shared by all genotypes may represent errors in the draft chicken assembly.

We searched for an inversion associated with Rose-comb using whole-genome resequencing of a 3.9 kb mate-pair library because this approach should precisely predict the location of any inversion breakpoints. The library was prepared from a pool of eight Rose-combed males from the Le Mans breed, all presumed to be homozygous *Rose-comb*. The library was sequenced to 1× coverage. Bioinformatic analysis of the data revealed aberrant mate pairs consistent with an inversion (Figure 2). Most aberrant reads (n = 22) indicated a 7.38 Mb inversion with breakpoints located approximately at 16.50 Mb and 23.88 Mb. However, three aberrant mate pairs connected the 16.50 Mb region with a region at 23.79 Mb, not consistent with a single inversion in all eight Rose-combed individuals included in the pool (Figure 2).

**Figure 3:**
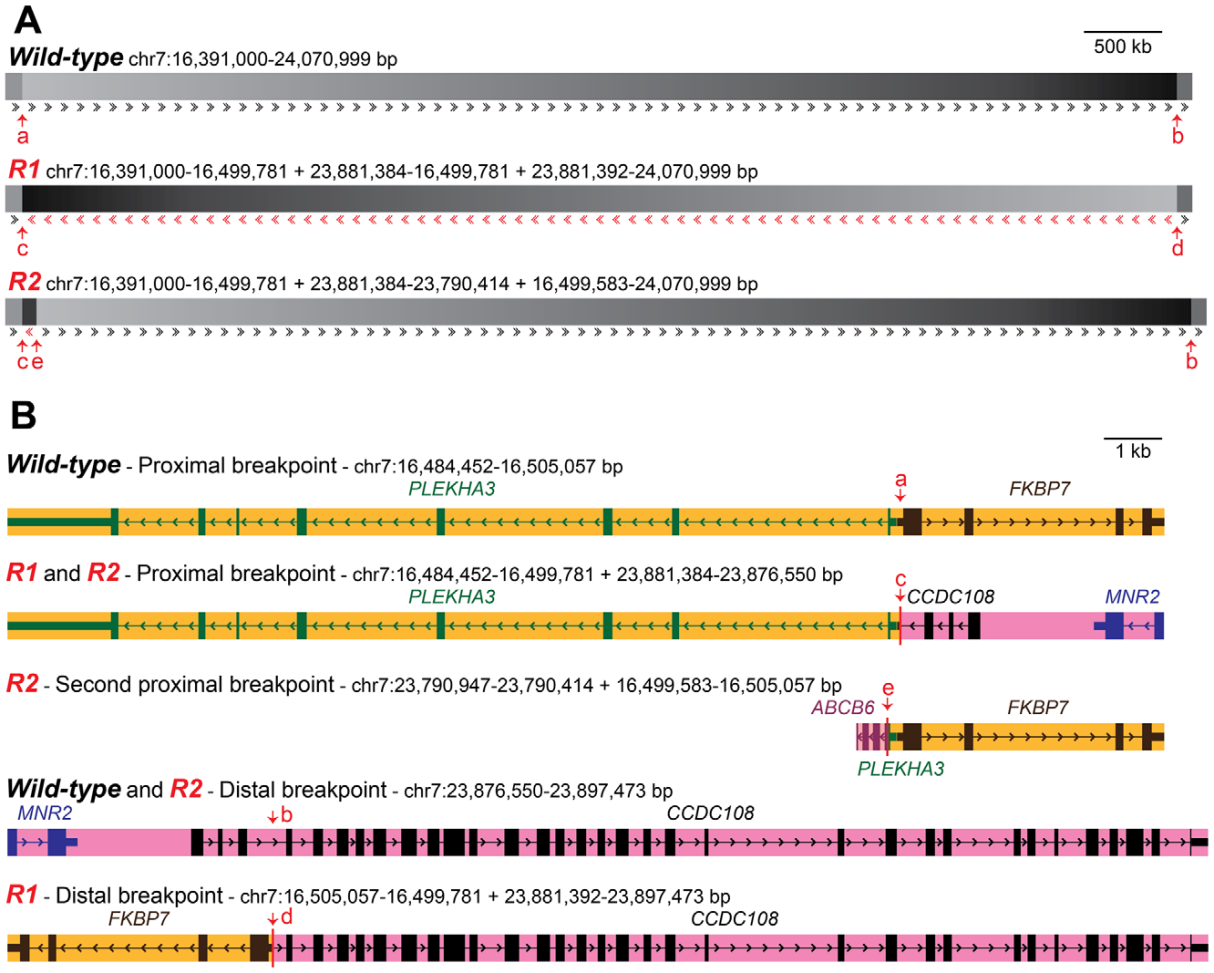
Organization of wild-type and *Rose-comb* chromosomes and description of inversion and duplication breakpoints. (A) Constitution of the two *Rose-comb* alleles, *R1* and *R2*, in relation to the organization of the wild-type (*r*) chromosome 7 in chickens. Sequence orientation in relation to the wild-type chromosome is indicated by arrows. Duplicated sequence in *R2* (chr7:23,790,414–23,881,384 bp) is in reverse orientation, apart from 198 bps (chr7:16,499,583–16,499,781 bp) flanking the inverted segment. Breakpoint locations are indicated by arrows (a–e). Breakpoints for the *R1* inversion are at 16,499,781 and 23,881,384-23,881,392 bp in the wild-type sequence. Additional breakpoints for the *R2* duplication are at 16,499,583 and 23,790,414 bp. (B) Organisation of genes in the five different chromosomal configurations associated with *Rose-comb*. Breakpoint locations are indicated with red arrows. mRNAs with accession numbers XM_422054.2, NM_204929.1, CR353563.1 and AJ719903.1, as well as EST sequences CD218766.1, BG713529.1 and DR426188.1 were used to define the genes illustrated. The copy of *ABCB6* that occurs at the second proximal breakpoint unique to the *R2* chromosome, is 5′ truncated from the duplication event, and appears 3′ truncated due to a gap in the assembly. An intact full length copy of this gene is expected to occur at its native chromosomal position (around 23.79 Mb) on *R1*, *R2* and *r* chromosomes.

PCR analysis of genomic DNA from Rose-combed individuals confirmed the inversion breakpoints at nucleotide positions 16,499,781 bp and 23,881,384–23,881,392 bp (Figure 3). A 628 bp gap is predicted around 16.50 Mb in the chicken galGal3 assembly. However, sequencing across the gap in the reference sequence bird (red junglefowl female from the UCD-001 line) revealed that the gap and an additional 87 bp that together constitute the chr7:16,499,808–16,500,522 bp region must be an assembly artefact; the correct sequence of this region has been submitted to GenBank with the accession number JN942757.

The presence of the inversion had an almost perfect association with the Rose-comb phenotype in a PCR-based screen of a large number of individuals from different breeds. No unambiguously single- or Pea-combed individual (n = 679) carried any of the breakpoints, as expected, and almost all Rose- or walnut-combed chickens (n = 872) carried both the 16.50 Mb breakpoint and the 23.89 Mb breakpoint (Table 2). However, some individuals from five breeds, that had an unambiguous Rose-comb, (n = 45) showed an aberrant pattern, scoring positive for the 16.50 Mb breakpoint but not for the 23.89 Mb breakpoint (Table 2). Therefore we postulated that these birds might carry a second *Rose-comb* allele, *R2*, that evolved from the original *Rose-comb* allele (*R1*) by a second rearrangement. Such an event would also be consistent with the presence of the aberrant mate-pair reads connecting the 16.50 Mb and 23.79 Mb regions (Figure 2). PCR amplification confirmed the existence of *R2*, and the results showed that the allele must have originated by a recombination event between the wild-type allele at position 16.50 Mb and the *R1* allele at position 23.79 Mb in the inverted region (Multimedia S1). The consequence of this recombination event is that *R2* does not carry the entire inversion but instead has two duplicated segments, one 91 kb fragment (23,790,414–23,881,384 bp) that represents a remaining fragment of the inverted region together with a small duplicated fragment of 198 bp (16,499,583–16,499,781 bp) that is present on both sides of the 91 kb duplication (Figure 3). Genotyping the eight resequenced Le Mans males revealed an allele composition of 4 *R1*, 9 *R2*, and 3 *r*, explaining why a large number of mate pairs over the breakpoint located at 16.50 Mb was found, as that is present in both *R1* and *R2*, while few reads spanning the breakpoint at 23.89 Mb were found, as that is only present in *R1*. The genome assembly is rich in gaps around 23.79 Mb, making it difficult to map reads that span the breakpoint only found in R2, explaining the low number of mapped reads spanning that breakpoint.

**Table 2:** Genotyping of the *Rose-comb* locus in various chicken breeds.

		Genotype					
Breed	Phenotype	*rr*	*R1R1*	*R2R2*	*R1R2*	*R1r*	*R2r*
Aijiao Yellow	Single	10	-	-	-	-	-
Alsacienne	Rose	-	1	4	1	-	-
Ameraucana	Pea	1	-	-	-	-	-
Anka	Single	10	-	-	-	-	-
Araucana	Pea	4	-	-	-	-	-
Ayam Cemani	Single	4	-	-	-	-	-
	Pea	3	-	-	-	-	-
Baier	Single	10	-	-	-	-	-
Beijing Fatty	Single	10	-	-	-	-	-
Bian	Rose	-	-	-	-	7	-
	Single	18	-	-	-	-	-
Brahma	Pea	4	-	-	-	-	-
Chahua	Single	10	-	-	-	-	-
Charollaise	Rose	-	5	-	-	1	-
Chongren Ma	Single	10	-	-	-	-	-
Cobb	Single	10	-	-	-	-	-
Cochin	Single	3	-	-	-	-	-
Crossbred Layer	Single	4	-	-	-	-	-
	Pea	7	-	-	-	-	-
Dagu	Single	10	-	-	-	-	-
Dongxiang Green Eggshell	Single	10	-	-	-	-	-
Dorking	Single	2	-	-	-	-	-
Faverolle	Single	2	-	-	-	-	-
Geline de Touraine	Single	4	-	-	-	-	-
Gushi	Single	10	-	-	-	-	-
Guyuan	Rose	-	10	-	-	55	-
	Single	5	-	-	-	-	-
Hamburg	Rose	-	1	-	-	3	-
Henan Game	Walnut	-	7	-	-	61	-
	Pea	113	-	-	-	-	-
Huiyang Beard	Single	10	-	-	-	-	-
Icelandic	Rose	-	3	-	-	9	-
	Single	28	-	-	-	-	-
	Undetermined	-	2	-	-	9	-
INRA F_0_s	Single	16	-	-	-	-	-
	Rose	-	-	-	-	2	-
INRA F_1_s	Rose	-	-	-	-	16	-
	Single	15	-	-	-	-	-
INRA resource	Rose	-	-	6	3	28	5
	Single	72	-	-	-	-	-
	Abnormal	-	2*	-	-	37*	-
Jinhu Wu	Rose	-	33	-	-	111	-
	Single	36	-	-	-	-	-
Kuaida Wu	Rose	-	38	-	-	105	-
	Single	36	-	-	-	-	-
Langshan	Single	10	-	-	-	-	-
Le Mans	Rose	-	1	2	2	-	3
Luyuan	Single	10	-	-	-	-	-
New Hampshire	Single	1	-	-	-	-	-
Oravka	Rose	-	6	1	7	2	4
Orlov	Variable	7	-	1	3	4	5
Orpington	Single	1	-	-	-	-	-
Plymouth Rock	Single	3	-	-	-	-	-
Poltava Clay	Variable	1	-	7	4	4	4
Qingyuan Ma	Single	10	-	-	-	-	-
Red Junglefowl	Single	13	-	-	-	-	-
Sebright	Rose	-	4	-	-	-	-
Shiqiza	Single	10	-	-	-	-	-
Shouguang	Single	10	-	-	-	-	-
Silkie	Walnut	-	5	-	-	2	-
	Rose	-	136	-	-	183	-
	Single or Pea	42	-	-	-	-	-
Sussex	Single	2	-	-	-	-	-
Tibetan	Single	10	-	-	-	-	-
Wahui	Single	10	-	-	-	-	-
Wenchang	Single	10	-	-	-	-	-
Westfälischer Totleger	Rose	-	13	-	3	4	-
White Leghorn	Single	10	-	-	-	-	-
White Rock	Single	10	-	-	-	-	-
Wyandotte	Rose	-	2	-	-	2	-
Xianju	Single	10	-	-	-	-	-
Xiaoshan	Single	10	-	-	-	-	-
Youxi Ma	Single	10	-	-	-	-	-
Yurlov	Rose	-	-	12	-	-	8
Total		687	269	33	23	645	29

Birds labelled with an asterisk were initially assumed to have a single-comb, but genotyping revealed that they carry the *R1* allele, a thorough examination of the comb phenotype revealed aberrant comb shape. Further information is given in Figure S1.

**Figure 4:**
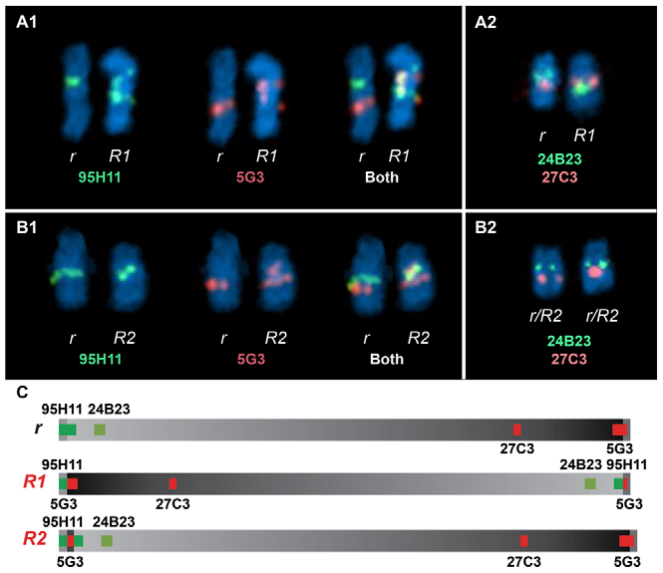
Two-colour FISH staining of metaphase chromosomes using BACs mapped to GGA7. (A1) Staining from a heterozygous *R1r* bird reveals two separate localisations for CH261-95H11 and CH261-5G3 when comparing *r* Chr7 to *R1* Chr7. (A2) The order reversal of BW27C3 and TAM32-24B23 between *r* Chr7 and *R1* Chr7 clearly demonstrates a large inversion. Staining from a heterozygous *R2r* bird reveals the same localisations obtained for CH261-95H11 (B1), TAM32-24B23 and BW27C3 (B2) both *r* Chr7 and *R2* Chr7, with CH261-5G3 showing an additional localisation on *R2* Chr7 (B1), consistent with a translocated duplication of a segment from the 23.88 MB region to the 16.50 MB region. A slight spatial separation for CH261-95H11, consistent with the insertion of the translocated duplication, can be observed on *R2* Chr7 (B1).

FISH analysis using four different BAC clones was employed to confirm the existence of two distinct *Rose-comb* alleles (Figure 4; Figure S3). The BAC clones CH261-95H11 and CH261-5G3 span the inversion breakpoints at 16.50 Mb and 23.88 Mb, respectively. BAC clones TAM32-24B23 and BW27C3 targeted regions within the inversion. A staining of a metaphase spread from an *R1r* heterozygote confirmed the presence of a large inversion on chromosome 7. The FISH staining of an *R2r* heterozygote metaphase spread was consistent with an altered organization, as the BACs CH261-95H11, TAM32-24B23 and BW27C3 showed indistinguishable staining for both *R2* and *r* chromosomes, with only two aberrant patterns obtained, one for BAC clone CH261-5G3 that confirmed the translocated duplication of a segment from the 23.88 MB region to the 16.50 MB region, and the other a slight spatial separation of BAC clone CH261-95H11, consistent with the insertion of the translocated duplication (Figure 4).

Previous studies of the Rose-comb phenotype have established that it involves both an altered comb morphology, showing dominant inheritance, and reduced male fertility, showing recessive inheritance. The presence of two different *Rose-comb* alleles facilitates the elucidation of the causal relationship between the observed chromosomal rearrangement and these two different aspects of the Rose-comb phenotype. No obvious phenotypic differences in comb morphology were observed amongst birds carrying *R1* and *R2*, matched for breed and genetic background (Figure S1). Thus the critical genetic lesion causing the Rose-comb morphology must be located at the 16.50 Mb breakpoint including the 91 kb segment transferred from the 23.79–23.88 Mb region, because this is the only alteration present in both *R1* and *R2*.

The proximal breakpoint at 16.50 Mb is located in the 5′UTR of *FKBP7* (FK506 binding protein 7) gene, 72 bp upstream of its start codon. Only 9 bp separate the 5′UTRs of *FKBP7* and *PLEKHA3* (Pleckstrin homology domain containing, family A-phosphoinositide binding specific-member 3), placing the breakpoint 42 bp from the 5′UTR, and 150 bp from the start codon of *PLEKHA3* (Figure 3B, Multimedia S1). This rearrangement may alter regulation of *PLEKHA3* and *FKBP7* in both *R1* and *R2* alleles.

The second proximal breakpoint, unique to *R2*, where an inverted duplicated 23.88-23.79 Mb region is joined with a duplicated 198 bp fragment at 16.50 Mb results in the duplication of a part of the ABCB6 (ATP-binding cassette, sub-family B (MDR/TAP), member 6) gene. ABCB6 has not been adequately annotated in the chicken genome, but this translocated duplication does not involve the first few exons, judging from annotated Expressed Sequence Tags (ESTs). Additionally, the region in which it is located is riddled with gaps in the genome assembly, causing the 3′ region of the EST range of the gene to appear truncated. The breakpoint seems to be close to the 3′ end of what may be exon 5 or 6, duplicating the very end of that exon and the remainder of the gene. This exon fragment copy is in its novel genomic context situated only 8 bp from the duplicated first exon of PLEKHA3. Whilst this could result in a hybrid transcript, the fact that an intact copy of ABCB6 is present on *R1*, *R2* and *r* chromosomes, as well as the presence of the duplicated segment being unique to *R2*, make it an unlikely candidate for the Rose-comb phenotype.

The distal breakpoint at 23.88 Mb is located in intron 3 of *CCDC108* (coiled-coil domain containing 108). This breakpoint disrupts *CCDC108* and transfers the first three exons to the proximal breakpoint present in both *R1* and *R2*. However, due to the intact nature of *CCDC108* in *R2* it was excluded as causative for the altered comb morphology. The neighbouring gene *MNR2* (MNR2 homeodomain protein), located only 3 kb from the inside of the distal inversion breakpoint, is also transferred to the near vicinity of the proximal breakpoint in *R1* and *R2*. The translocation of the transcription factor MNR2 to a novel genomic context was considered the best candidate for causing altered comb morphology in *Rose-comb* as it belongs to the Mnx-class of homeodomain proteins which act as transcriptional repressors and specifiers of cell identity [26]. Furthermore, hyaluronan (HA), a major component of the extracellular matrix and the comb in chickens, shows strong accumulation around early MNR2-expressing neurons [27].

### Expression analysis using comb tissue from wild-type, Rose-combed, Pea-combed, and walnut-combed embryos

**Figure 5:**
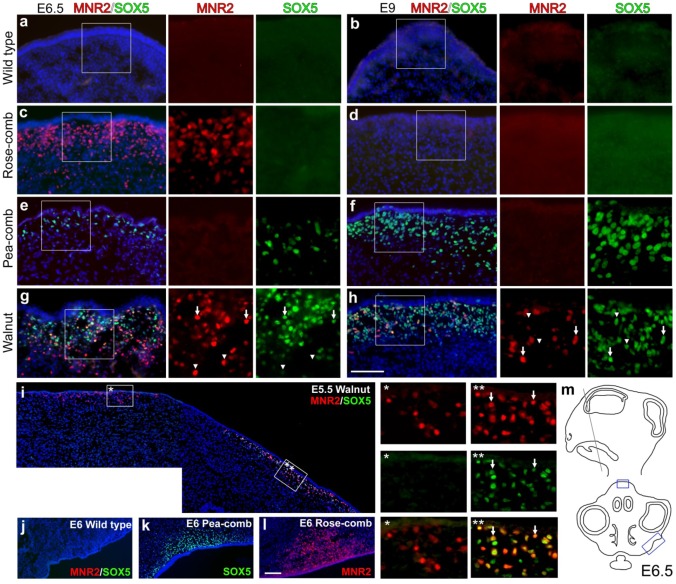
Immunohistochemical labelling of MNR2 and SOX5 in various comb tissues. Wild-type single-comb (a, b), Rose-comb (c, d), Pea-comb (e, f) and walnut-comb (g, h) sections from embryonic day (E) 6.5 (a, c, e, g) and 9 (b, d, f, h) were labelled against MNR2 and SOX5. Nuclei are visualized by DAPI. Boxed regions are shown magnified as single colour. Arrows in (g) and (h) indicate double labelled cells whereas arrowheads indicate single labelled cells. (i) E5.5 walnut comb. The two framed regions are shown magnified and arrows indicate double labelled cells. (j–l) The prospective wattle region for wild-type single-comb, Pea-comb and Rose-comb. (m) Schematic view of an E6.5 head where boxes indicate the regions of the comb depicted in (a–h) and wattles depicted in (j–l). Scale bar equals 100 µm in (h) and is valid for (a–h) and 100 µm in (l) valid for (i–l).

Expression analysis of comb tissue from early embryos (single-combed wild-type and *R1R1* homozygotes) by RT-PCR revealed that *PLEKHA3* and *FKBP7* were expressed in both wild-type and homozygous *Rose-comb* tissue, whereas *CCDC108* and *MNR2* were expressed in Rose-combed but not in wild-type embryos (Figure S4). The ectopic *MNR2* expression was restricted to days E6–E12 of embryonic development. This suggested that the Rose-comb phenotype might be due to ectopic expression of the MNR2 homeodomain protein as a copy of *MNR2* is translocated close to the 16.50 Mb breakpoint in both *R1* and *R2*. To further explore this possibility, as well as shed light on the interaction between *Rose-comb* and *Pea-comb* causing the walnut-comb phenotype, we performed immunohistochemical staining using an anti-chicken MNR2 antibody and an anti-human SOX5 antibody (previously used in our characterization of *Pea-comb* [21]) in single-combed wild-type, Rose-combed, Pea-combed and walnut-combed embryos (Figure 5). This analysis revealed transient ectopic expression of MNR2 in Rose-combed embryos, consistent with the results of the RT-PCR analysis. Striking MNR2 expression was observed in a layer of mesenchymal cells located in the area where the comb is developing at day E6.5 but not at E9 (Figure 5C and 5D). This pattern of transient ectopic expression resembles that previously reported for SOX5 in Pea-combed embryos, where expression is weak at day E5, strong at E9 (Figure 5E and 5F) and not present at day E12 [21].

Walnut-combed embryos showed transient ectopic expression of both MNR2 and SOX5 as expected from their genotype (Figure 5G–5H). The ectopic expression of MNR2 and SOX5 overlapped only partially, with peak expression occurring first for MNR2. The data revealed ectopic expression of MNR2 and SOX5 in the same cell type as well as MNR2-SOX5 coexpression within individual cells (Figure 5I). Furthermore, MNR2 expression was observed at E9 together with SOX5 (Figure 5H) but this was not the case in the absence of SOX5 expression (Figure 5D) suggesting a potential positive interaction between the *Pea-comb* allele of SOX5 and the *Rose-comb* allele of MNR2. Interestingly, at day E6 MNR2 and SOX5 also showed ectopic expression in Rose-combed and Pea-combed birds, respectively, in mesenchyme present in the region where the wattles develop (Figure 5J–5L). The ectopic expression of MNR2, with its transcriptional repression activities, is likely to change the identity of the mesenchyme underlying both the comb and wattles. Seminal work on comb primordium development has shown that the comb shape is directly dependent on instructive signals derived from the underlying mesenchyme/dermal structures [28]–[30]. This suggests that the comb and wattle tissue share a common developmental pathway and that the structural variants underlying Rose-comb and Pea-comb activate the expression of MNR2 and SOX5, which both modulate or intercept this pathway. However, wattle phenotype is not altered by the *Rose-comb* mutation as it is by the *Pea-comb* mutation (Figure 1).

### 5′RACE analysis of transcripts initiated from the vicinity of the inversion breakpoints

5′RACE analysis was performed using tissue from early embryonic comb and from adult testis for the three genes located close to the two *R1* inversion breakpoints. Results are summarized in Figure S5. The samples were from single-combed wild-type birds and *R1R1* homozygotes. A full length PLEKHA3 transcript (denoted PLEKHA3a in Figure S5) was expressed in both tissues and in both genotypes. A PLEKHA3-CCDC108 hybrid transcript (PLEKHA3b) corresponding to exons 1 and 3 from CCDC108 and exons 2–8 from PLEKHA3 was found in Rose-comb testis (Figure S6). The FKBP7 transcripts showed no difference between genotypes but different splice forms were expressed in comb and testis. Full-length CCDC108 transcripts were only found in wild-type testis (Figures S5 and S6), whereas two very similar hybrid transcripts lacking the first three exons of CCDC108 were expressed in both *R1R1* testis and comb tissue (Figures S5 and S6).

### Analysis of male fertility in *Rose-comb* homozygotes

*Rose-comb* is associated with reduced male fertility in homozygotes due to low sperm motility [12], [17], [18]. However, as *R1* is the more common *Rose-comb* allele (Table 2) we wanted to investigate if the fertility effect is also associated with the *R2* allele discovered in the present study. A preliminary study to address this question was carried out by mating single-combed and Rose-combed roosters of several genotypes (*R1R1, R2R2, R1r* and *rr*) to single-combed and Rose-combed hens. The data were consistent with reduced male fertility in *R1R1* males, as expected, but there were no signs of reduced fertility in *R2R2* homozygotes (Text S2; Table S1). Thus, the deleterious effect on male fertility appears to be restricted to the *R1* allele, suggesting that the lesion causing this phenotypic effect is located at the 23.88 Mb breakpoint (Figure 3). The obvious candidate for this effect is the disruption of the *CCDC108* transcript that leads to the expression of a truncated transcript in testis (Figures S5 and S6).

## Discussion

The present study illustrates several striking features of the genetic diversity present in domestic animals. Firstly, the *R2* allele exemplifies the evolution of alleles by two or more consecutive mutations. Other examples include the *Dominant white* allele in pigs which involves a 450 kb duplication encompassing the entire *KIT* gene combined with a splice mutation in one of the duplicated copies [31] and black spotting in pigs which is determined by the combined effects of two mutations in *MC1R*, a missense mutation associated with black colour and a somatically unstable two base-pair insertion [32]. Secondly, it represents a new example of how structural rearrangements have contributed to rapid phenotypic evolution observed in domestic animals [33], [34]. The majority of the structural changes reported to be associated with phenotypic effects, like the effects of *R1* and *R2* on comb morphology, constitute cis-acting regulatory mutations. The altered configurations of regulatory elements on the rearranged chromosome lead to altered gene expression patterns. It appears plausible that structural rearrangements similar to those that affect comb development in chickens have also contributed to phenotypic evolution in natural populations, including human populations.

There are several mechanisms by which genomic rearrangements are thought to occur, involving either double strand break repair via primarily non-homologous mechanisms or homology mediated replication and recombination based processes [35], [36]. At the *R1* and *R2* 16.50 Mb breakpoint there is only a single base pair sequence overlap, while at the *R1* 23.88 Mb breakpoint there is a 7 bp overlap with one mismatch. Although it is not possible to determine the exact mechanism by which the original inversion occurred, it is likely that microhomology at the 23.88 Mb breakpoint was involved in the generation of the *R1* allele. The generation of the *R2* allele occurred by recombination between a wild-type chromosome and an *R1* chromosome (Multimedia S1). On the *wild-type* chromosome this event occurred 198 bp upstream of the 16.50 Mb breakpoint, on the *R1* chromosome the recombination event occurred 91 kb into the inversion from the 16.50 Mb breakpoint. This resulted in the duplication of the 91 kb portion of the *R1* chromosome, including the *R1* proximal breakpoint and the 199 bp fragment of the wild-type chromosome, effectively inserting the duplicated sequence at 16.50 Mb into a wild-type chromosome. Analysis of the *R2* recombination event breakpoint shows 2 bp of sequence homology, again suggesting that sequence microhomology was likely involved. That both events introduced breakpoints around 16.50 Mb, spaced only 198 bp apart, suggests that some characteristic of this region may predispose it to such events.

More than half of the loci identified in human genome-wide association analyses do not overlap coding regions [37], implying that they reflect regulatory polymorphisms. The present study illustrates how challenging it can be to reveal the molecular mechanism underlying even a simple monogenic trait in a model organism such as the chicken. We were able to demonstrate transient ectopic expression of MNR2 during a narrow period of embryonic development because immunohistochemistry provided the spatial resolution allowing the detection of ectopic expression in a subset of cells within the affected tissue (Figure 5).

We postulate that the well-established association between the Rose-comb phenotype and reduced sperm motility is restricted to the *R1* allele and that it is caused by the disruption of the *CCDC108* transcript. The predicted CCDC108 protein sequence in chickens shows 49% amino acid identity with human CCDC108 (HomoloGene:28093; www.ncbi.nlm.nih.gov). CCDC108 has an unknown function, according to the UNIPROT database (www.uniprot.org/uniprot/Q6ZU64), it is a single pass membrane protein composed of 1925 amino acids, containing one MSP (major sperm protein) domain. The MSP domain is present in major sperm proteins and in sperm specific proteins (SSPs) found in various nematodes as well as in the mammalian Motile sperm domain-containing proteins-1, -2 and -3 (MOSPD1-3). All MSP, SSP and MOSPD proteins are small, with a size range of 107–518 amino acids, and thus much smaller than CCDC108. Mouse Ccdc108 is expressed in testis and shows differential expression during progression of spermatogenesis [38]; expression is absent in juvenile mice but is turned on when male mice reach sexual maturity (www.ncbi.nlm.nih.gov; GEO profiles GDS606/164004_at/Ccdc108/Mus musculus). Furthermore, by using reciprocal best-hit protein BLAST searches, putative CCDC108 orthologs can be found in many organisms, including in deep branches on the tree of life. One such orthologous protein has been annotated in *Chlamydomonas* algae as an axonemal protein named Flagellar Associated Protein 65 (FAP65) [39]. FAP65 expression is strongly induced after deflagellation, when cells regenerate their flagella. This sequence homology suggests that CCDC108 is part of the sperm flagellum and thus that a disruption of CCDC108 function may lead to sperm motility defects as observed in Rose-comb *R1R1* homozygotes. This study establishes *CCDC108* as a candidate gene for sperm motility disorders in humans.

The present study strongly suggests that Rose-comb and Pea-comb are caused by transient ectopic expression of two potent transcription factors, MNR2 and SOX5, respectively. However, at present we cannot exclude the possibility that altered expression levels of *FKBP7* or *PLEKHA3*, located on either side of the proximal breakpoint, may have some impact on the comb phenotype. However the ectopic MNR2 expression in the developing embryo is by far the most striking molecular consequence of the Rose-comb inversion. The exact downstream mechanisms leading to the altered comb morphology remain undetermined. Comb tissue is composed of layers of epidermis, dermis and central connective tissue comprising primarily collagen and hyaluronan (HA) [40]. The previous report that there is a strong accumulation of HA around early MNR2-expressing neurons [27] may be relevant for the Rose-comb phenotype, but perhaps more importantly, MNR2 acts as a repressor and specifier of cell identity [26]. SOX5 also has an established functional role that makes sense in relation to the altered comb morphology observed in Pea-combed and walnut-combed birds. SOX5 contributes to chondrogenesis, together with SOX6 and SOX9 it activates specific genes during embryonic cartilage formation [41], and has a repressive role in oligodendrogenesis during neural development [42]. A fascinating observation is that both the *Rose-comb* mutations and the *Pea-comb* mutation give rise to ectopic expression in the area of the developing comb, leading to altered comb morphology in both mutants, as well as in the wattle area, which only leads to altered morphology in birds carrying *Pea-comb* (Figure 1). The fact that the transient ectopic expression of MNR2 and SOX5 apparently occurs in the same population of mesenchymal cells, and with at least partially overlapping expression in individual cells in the comb primordium, provides a reasonable explanation of why the combined effect of the two mutations leads to the formation of the walnut-comb. Thus, 104 years after Bateson and Punnett [2] reported the first example of epistatic interaction between genes, we can now provide a molecular explanation for their seminal observation.

## Materials and Methods

### Ethics statement

All animal work has been conducted according to relevant national and international guidelines.

### Animals

Linkage mapping was carried out using a pedigree consisting of two heterozygous *R1r* male parentals, each mated with eight homozygous *rr* females, resulting in 383 progeny segregating for *Rose-comb*. The Rose-combed roosters were from an INRA (French National Institute for Agricultural Research) resource population, with the *R1* allele having been derived from the French breed Charollaise. The wild-type single-combed hens were from another INRA resource population line.

DNA samples from various chicken breeds were genotyped for the *R1*, *R2* and *wild-type* alleles. These included samples collected as part of the AvianDiv project [43], from resource populations at INRA, and 31 different breeds of Chinese chickens collected by Institute of Poultry Science, Chinese Academy of Agricultural Sciences. Blood samples from privately owned Icelandic chickens were obtained at eight different locations in the South-West of Iceland by a veterinarian with permission from owners. Genomic DNA from the reference red junglefowl bird was kindly provided by Dr. J.B. Dodgson.

### Linkage analysis

Linkage analysis was performed by genotyping two microsatellites and nine SNPs from chromosome 7 using standard procedures. Custom TaqMan SNP Genotyping Assays (Applied Biosystems) were designed by ABI, other primers were designed with the Primer3Plus webtool (http://www.bioinformatics.nl/cgi-bin/primer3plus/primer3plus.cgi). See Table S2 for primer and probe sequence information. Linkage analysis was performed with the Crimap software (version 2.4) [44].

### Whole-genome resequencing

DNA from eight Rose-combed males from the Le Mans breed, all presumed to be homozygous for *Rose-comb*, were pooled. Whole genome resequencing data from a pool of Rose-combed Silkie chickens, and another pool of single-combed White Leghorns were obtained at later time points and included in this study to verify the results obtained from the Le Mans pool. A sequencing library was generated for the Le Mans sample using a Mate-pair SOLiD3 protocol and sequenced on SOLiD v.3 (Life Technologies, Carlsbad, USA). The White Leghorn library was generated using a Mate-pair SOLiD3+ protocol and sequenced on SOLiD 3+. The Silkie Library was generated using a Mate-pair SOLiD5500 protocol and sequenced on SOLiD5500XL. The Le Mans, White Leghorn and Silkie reads (2×50 bp mate-pair reads) were mapped to the chicken genome (WUGSC 2.1/galGal3) reference assembly using the software CoronaLite v0.4r2, Bioscope v1.0.1 and LifeScope v2.0, respectively, with average insert sizes estimated as approximately 3.9, 3.1 and 2.6 kb and average read depth approximately 1×, 10× and 20× over the chicken genome. Mapping distances between mate-pairs were used to detect structural variations in relation to the reference assembly. All library kits, alignment software and massively parallel sequencing equipment were used according to the manufacturer's instructions (Life Technologies, Carlsbad, USA).

### Fluorescent *in situ* hybridisation (FISH)

Heterozygous Rose-combed embryos (*R1r* and *R2r*) were produced from parental stock maintained at INRA. The *R1* allele originated from Belgian Barbu d'Anvers and the *R2* allele from French Alsacienne. BAC clones were chosen considering their position in the chicken genome sequence (Table S3). BW27C3 comes from the Wageningen library [45]. TAM32-24B23 was ordered from TAMU (Texas A&M BAC Libraries, USA). CH261-5G3 and CH261-95H11 were ordered from the Children's Hospital Oakland Research Institute in Oakland (CHORI), California, USA. BAC clones were grown in LB medium with 12.5 µg/ml chloramphenicol according the instructions of the providers. The DNA was extracted using the Qiagen plasmid midi kit.

FISH was carried out on metaphase spreads obtained from fibroblast cultures derived from 7 days old embryos, arrested with 0.05 µg/ml colcemid (Sigma) and fixed by standard procedures. The FISH protocol is derived from Yerle *et al*. [46]. Two-colour FISH was performed by labelling 100 ng of each BAC clone with alexa fluorochromes (ChromaTide Alexa Fluor 488-5-dUTP, Molecular probes; ChromaTide Alexa Fluor 568-5-dUTP, Molecular Probes) by random priming using the Bioprim Kit (Invitrogen). The probes were purified using spin column G50 Illustra (Amersham Biosciences). Probes were ethanol precipitated together and hybridised to the metaphase slides for 17 h at 37°C in the Hybridizer (Dako) after denaturation for 8 min at 72°C. Chromosomes were counterstained with DAPI in antifade solution (Vectashield with DAPI, Vector). The hybridised metaphases were screened with a Zeiss fluorescence microscope. A minimum of twenty spreads was analysed for each experiment. Spot-bearing metaphases were captured and analysed with a cooled CCD camera using Cytovision software (Applied Imaging, Leica-Microsystem). Images were formatted, resized and arranged for publication using Adobe Photoshop and Adobe Illustrator.

### PCR analysis of rearrangement breakpoints

A set of five PCR primers that together will amplify a series of specific bands over each of the five breakpoints was designed for genotyping the *R1*, *R2* and *r* alleles. Primer and protocol information are in Table S4. Gel image for the six possible genotypes is presented in Figure S7.

### RT–PCR analysis of tissue samples

Comb tissue was collected from homozygous (*R1R1*) and heterozygous (*R1r*) Rose-combed birds as well as homozygous (*rr*) single-combed wild-type birds. Comb tissue was sampled at embryonic (E) days 6, 7, 8, 9, 10, 11, 12, 15 and 19. Testis tissue was sampled from adult roosters at day 200. Samples from three birds of each type were collected and stored in RNAlater (Ambion). RNA was extracted using RNeasy Mini kit (Qiagen). cDNA was synthesized with 1 µg of RNA using oligo(T) primer. Primers spanning introns were used in RT-PCR. The 5′ and 3′ RACE were performed using GeneRacer Kit (Invitrogen).

### Immunohistochemistry

Homozygous Rose-combed Alsacienne, single-combed INRA resource population, homozygous Pea-combed Cheptel and heterozygous walnut-combed Alsacienne x Cheptel embryos were used. Heads from staged embryos were fixed in 4% paraformaldehyde in phosphate buffered saline (PBS) for one hour at 4°C. Fixed heads were incubated overnight in 30% sucrose in PBS at 4°C, embedded in OCT freezing medium (Tissue-Tek, Sakura), frozen and sectioned in a cryostat. Cross sections, 10 µm thick, were collected on glass slides (Super Frost Plus, Menzel-Gläser). The sections were rehydrated in PBS for 5 min and then blocked for one hour in PBS containing 1% fetal calf serum, 0.1% Triton-X and 0.02% Thimerosal. The antibodies MNR2 (Developmental studies hybridoma bank, 81.5C10) and SOX5 (Abcam, a_6226041) were diluted 1∶250 and 1∶1000 respectively in blocking solution and incubated on the slides overnight at 4°C. The secondary antibodies (Invitrogen) were incubated at room temperature for two hours at a 1∶1000 dilution in blocking solution. Samples were analysed using a Zeiss Axioplan2 microscope equipped with Axiovision software. Images were formatted, resized, enhanced and arranged for publication using Axiovision and Adobe Photoshop.

### URL

Information on the chicken genome sequence is available at http://www.genome.ucsc.edu.

### Accession numbers

The sequence data presented in this paper have been submitted to GenBank with accession numbers JN942757–JN942760, JN880446, JN880447, JQ004983, and JQ004984.

## Supporting Information

Figure S1 Phenotypic variability of Rose-comb in Icelandic chickens (A-W), Alsacienne (X), and INRA resource population (Y, Z, a, b). D, H, L, P and T are homozygous *R1R1*, X is homozygous *R2R2*, and all others are heterozygous *R1r*. All Icelandic chickens with phenotypic record that typed negative for the *R1* allele (n = 28) had a phenotypic single-comb, indicating that Pea- and Duplex-comb alleles do probably not segregate in the breed, or if they do, the frequency is very low. This leaves Rose-comb and Crest as the major known phenotypic traits affecting comb shape in the Icelandic chicken, which is very variable as seen in images (A-W). All INRA resource population birds assumed to be single-combed that typed positive for *R1* had a comb shape deviating from single-comb as exemplified by images (Y, Z, a and b). It is evident that there is an enormous variability in the phenotypic presentation of Rose-comb, with the traditional smooth and rough classical Rose-combs so well documented in the literature only representing a portion of the possible comb shapes that the *Rose-comb* mutation can give rise to. Additional variation in the vicinity of *MNR2* or at other loci is likely to contribute to this variability. Photos by Freyja Imsland (A-W), Michèle Tixier-Boichard (X) and David Gourichon (Y, Z, a, b).
(TIF)

Figure S2 Average heterozygosity of chicken chromosome 7 in different populations. An Illumina 60K SNP array was used to genotype 1271 birds from 15 breeds. 1830 SNPs from GGA7 were included in the analysis. Results for 67 Rose-combed (*R1R1*) birds from Henan Game, Jinhu Wu, Kuaida Wu and Silkie breeds are represented by a blue line. Results for 1204 wild-type (*rr*) birds from 15 breeds (Anka, Beijing Fatty, Chahua, Henan Game, Huiyang Beard, Jinhu Wu, Kuaida Wu, Langshan, Qingyuan Ma, White Rock, Red Jungle Fowl, Shiqiza, Silkie, Tibetan and Wenchang) are represented by a red line. The heterozygosity (H) was calculated for each locus as: H = 1-Σpi∧2, where pi is the frequency of the i-th allele for a given locus. Heterozygosity was assessed in 500-kb sliding windows.
(TIF)

Figure S3 Full FISHed metaphases for the *R1r* (A) and *R2r* (B) genotypes used to generate Figure 4. Chromosomes 7 (GGA7), labelled with fluorescent probes, are indicated by arrows. (A1) Staining from a heterozygous *R1r* bird reveals two separate localisations for CH261-95H11 and CH261-5G3 when comparing *r* Chr7 to *R1* Chr7. (A2) The order reversal of BW27C3 and TAM32-24B23 between *r* Chr7 and *R1* Chr7 clearly demonstrates a large inversion. Staining from a heterozygous *R2r* bird reveals the same localisations obtained for CH261-95H11 (B1), TAM32-24B23 and BW27C3 (B2) both *r* Chr7 and *R2* Chr7, with CH261-5G3 showing an additional localisation on *R2* Chr7 (B1), consistent with a translocated duplication of a segment from the 23.88 MB region to the 16.50 MB region.
(TIF)

Figure S4 RT-PCR analysis of *CCDC108*, *GAPDH*, *FKBP7*, *MNR2* and *PLEKHA3* using embryonic comb tissue from single-combed wild-type (*rr*) and Rose-combed (*R1r* and *R1R1*) chickens. E6–E19 represent embryonic days 6 to 19. M = molecular weight marker. H_2_O = negative control.
(TIF)

Figure S5 Schematic presentation of the 5′RACE products obtained using embryonic comb tissue and adult testis from single-combed wild-type and Rose-combed (*R1R1*) homozygotes. Three genes (*PLEKHA3*, *FKBP7* and *CCDC108*) located in the vicinity of the two *R1* inversion breakpoints were investigated. Red vertical bars represent inversion breakpoints. The chromosomal background colour code is consistent with the one used in Figure 3, yellow represents sequences from the 16.50 Mb region and pink sequences from the 23.88 Mb side. WC = wild-type comb tissue; WT = wild-type testis; RC = Rose-comb tissue; RT = Rose-comb testis.
(TIF)

Figure S6 RT-PCR analysis of different transcripts in testis. Homozygous single-combed wild-type (*rr*), heterozygous (*R1r*) and homozygous (*R1R1*) rose-combed animals were used. GAPDH was used as a positive control. The CCDC108 amplicons include exons 15–17, present in both wild-type and mutant transcripts (see Figure S5). The PLEKHA3 amplicons include exons 4–7, present in both PLEKHA3a and PLEKHA3b. Full length FKBP7 and MNR2 transcripts were detected in all three genotypes.
(TIF)

Figure S7 Gel images of electrophoresed PCR products, showing the results obtained with the diagnostic test detailed in Table S4, for the six different genotypes at the *Rose-comb* locus in chicken. Fragment sizes (in bp) as well as their association to different alleles are indicated to the right.
(TIF)

Multimedia S1 Origin of the *R1* and *R2* alleles of chicken chromosome 7 (GGA7), with depictions of gene arrangement at breakpoint locations. The *R1* allele arose by an inversion event, where 7.4 Mb of GGA7 were inverted. The *R2* allele arose by non-homologous recombination between wild-type *GGA7* and *R1*. Sequence orientation in relation to the wild-type chromosome is indicated by arrows. Duplicated sequence in *R2* (chr7:23,790,414–23,881,384 bp) is in reverse orientation, apart from 198 bps (chr7:16,499,583–16,499,781 bp) flanking the inverted segment. Breakpoints for the *R1* inversion are at 16,499,781 and 23,881,384–23,881,392 bp in the wild-type sequence. Additional breakpoints for the *R2* duplication are at 16,499,583 and 23,790,414 bp. The *Rose-comb* allelic series involves five different breakpoints, each with its own gene arrangement. The wild-type arrangement of both the 16.50 and 23.88 Mb breakpoints are shown first. Then that of the *R1*/*R2* arrangement of the 16.50 Mb breakpoint, after that the arrangement of the *R2* 23.79 Mb breakpoint and finally that of the *R1* 23.88 Mb breakpoint. mRNAs with accession numbers XM_422054.2, NM_204929.1, CR353563.1 and AJ719903.1, as well as EST sequences CD218766.1, BG713529.1 and DR426188.1 were used to define the genes illustrated. The copy of *ABCB6* that occurs at the 23.79 Mb breakpoint, unique to the *R2* chromosome, is 5′ truncated from the duplication event, and appears 3′ truncated due to an assembly gap. An intact full length copy of this gene is expected to occur at its native chromosomal position (around 23.79 Mb) on *R1*, *R2* and wild-type chromosomes.
(SWF)

Table S1 Fertility data for different matings involving Rose-comb chicken.
(PDF)

Table S2 Compilation of primer sequences used for the *Rose-comb* locus.
(PDF)

Table S3 Genomic coordinates of BACs used for FISH imaging. BAC BW27C3 has been screened for a genetic marker (MCW201) in the indicated location, but the precise extent of the BAC is unknown, as the ends have not been sequenced.
(PDF)

Table S4 Diagnostic PCR-based screening of breakpoints associated with the *Rose-comb* alleles.
(PDF)

Text S1 High-resolution mapping of the *Rose-comb* locus using a second pedigree.
(PDF)

Text S2 Test matings to assess male fertility.
(PDF)
