# Supplementary figures and images for: The Rose-comb Mutation in Chickens Constitutes a Structural Rearrangement Causing Both Altered Comb Morphology and Defective Sperm Motility
Source: PLoS Genet. 2012 Jun 28;8(6):e1002775. doi: 10.1371/journal.pgen.1002775 (PMC3386170; doi:10.1371/journal.pgen.1002775)

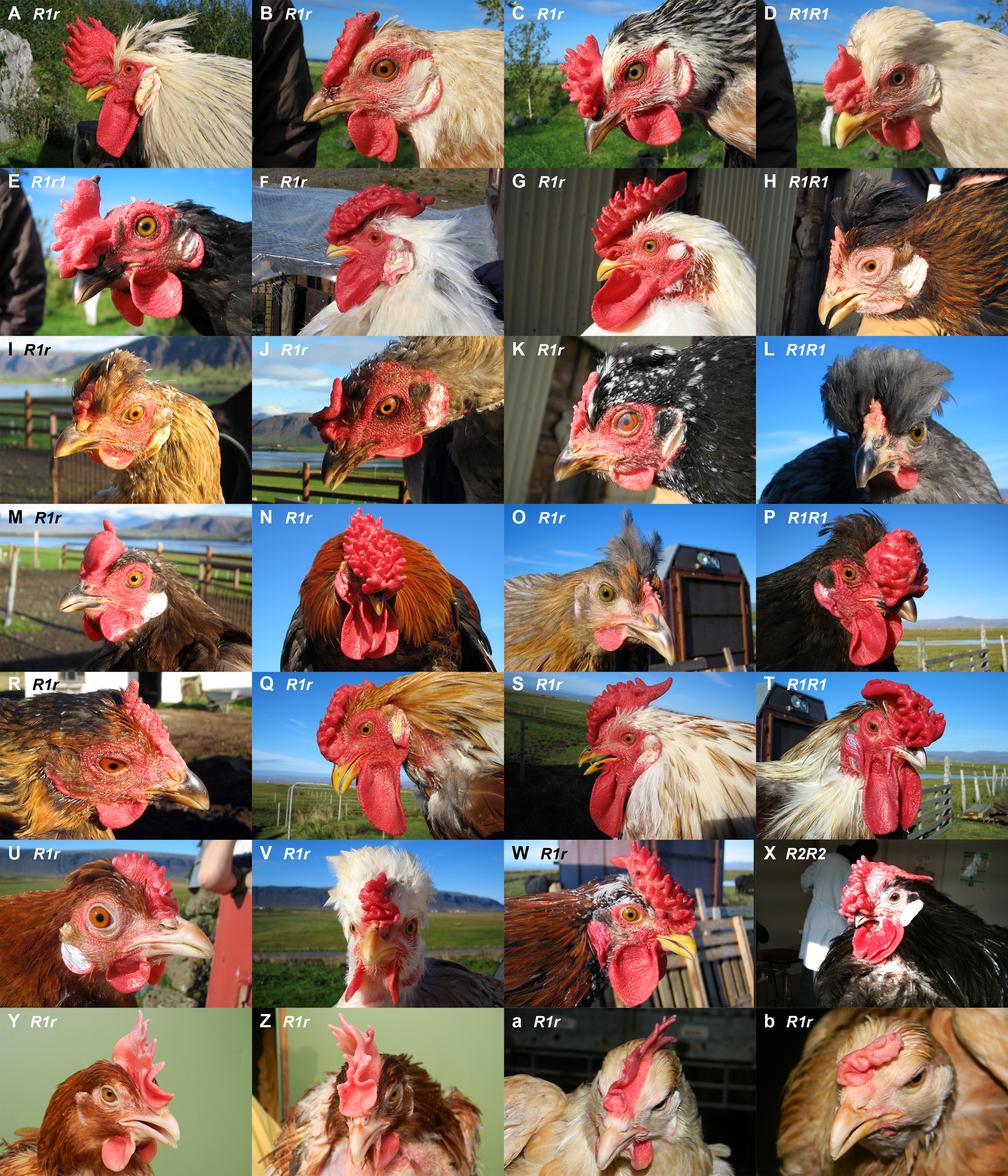

Supplement: Figure S1 — Phenotypic variability of Rose-comb in Icelandic chickens (A-W), Alsacienne (X), and INRA resource population (Y, Z, a, b). D, H, L, P and T are homozygous R1R1, X is homozygous R2R2, and all others are heterozygous R1r. All Icelandic chickens with phenotypic record that typed negative for the R1 allele (n = 28) had a phenotypic single-comb, indicating that Pea- and Duplex-comb alleles do probably not segregate in the breed, or if they do, the frequency is very low. This leaves Rose-comb and Crest as the major known phenotypic traits affecting comb shape in the Icelandic chicken, which is very variable as seen in images (A-W). All INRA resource population birds assumed to be single-combed that typed positive for R1 had a comb shape deviating from single-comb as exemplified by images (Y, Z, a and b). It is evident that there is an enormous variability in the phenotypic presentation of Rose-comb, with the traditional smooth and rough classical Rose-combs so well documented in the literature only representing a portion of the possible comb shapes that the Rose-comb mutation can give rise to. Additional variation in the vicinity of MNR2 or at other loci is likely to contribute to this variability. Photos by Freyja Imsland (A-W), Michèle Tixier-Boichard (X) and David Gourichon (Y, Z, a, b). (TIF) [file pgen.1002775.s001.tif]

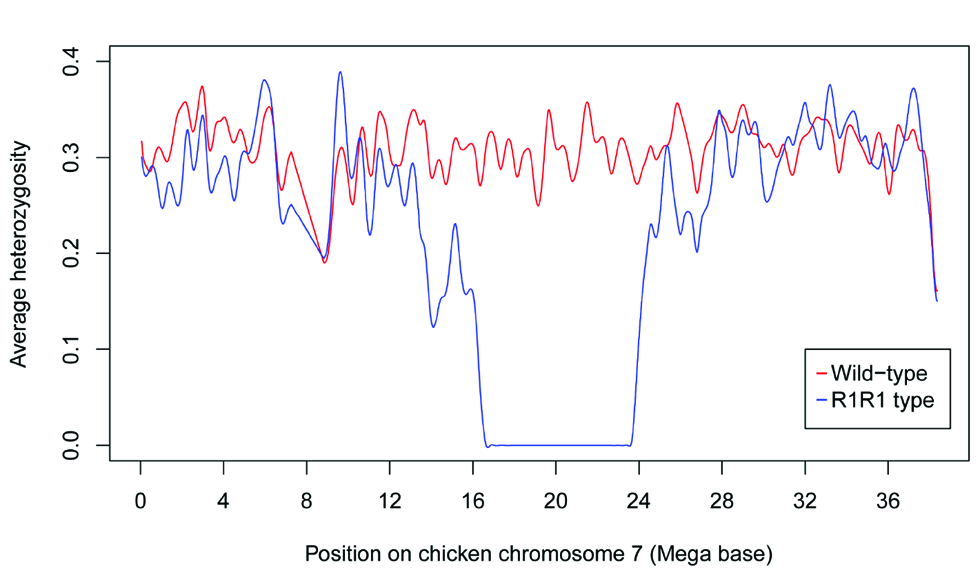

Supplement: Figure S2 — Average heterozygosity of chicken chromosome 7 in different populations. An Illumina 60K SNP array was used to genotype 1271 birds from 15 breeds. 1830 SNPs from GGA7 were included in the analysis. Results for 67 Rose-combed (R1R1) birds from Henan Game, Jinhu Wu, Kuaida Wu and Silkie breeds are represented by a blue line. Results for 1204 wild-type (rr) birds from 15 breeds (Anka, Beijing Fatty, Chahua, Henan Game, Huiyang Beard, Jinhu Wu, Kuaida Wu, Langshan, Qingyuan Ma, White Rock, Red Jungle Fowl, Shiqiza, Silkie, Tibetan and Wenchang) are represented by a red line. The heterozygosity (H) was calculated for each locus as: H = 1-Σpi∧2, where pi is the frequency of the i-th allele for a given locus. Heterozygosity was assessed in 500-kb sliding windows. (TIF) [file pgen.1002775.s002.tif]

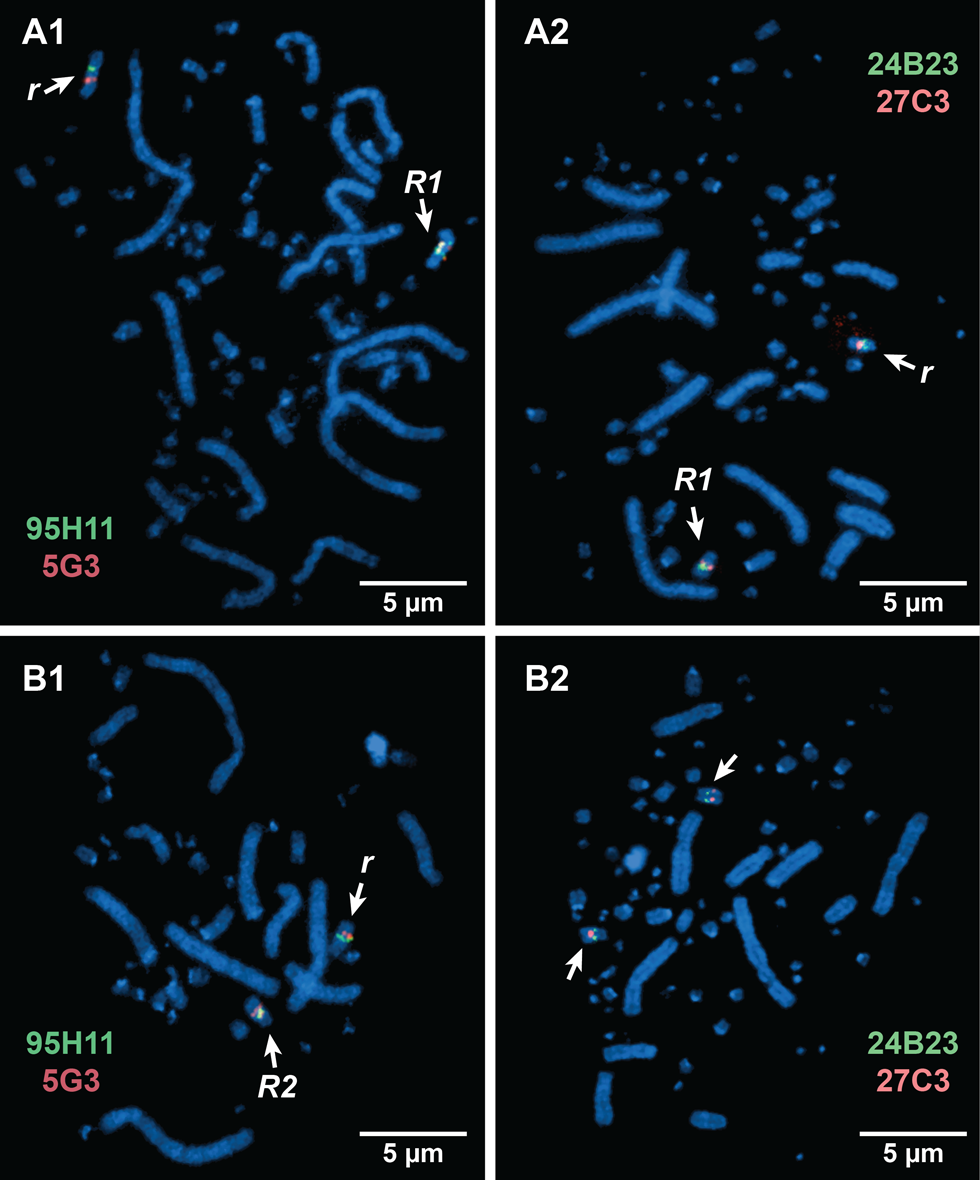

Supplement: Figure S3 — Full FISHed metaphases for the R1r (A) and R2r (B) genotypes used to generate Figure 4. Chromosomes 7 (GGA7), labelled with fluorescent probes, are indicated by arrows. (A1) Staining from a heterozygous R1r bird reveals two separate localisations for CH261-95H11 and CH261-5G3 when comparing r Chr7 to R1 Chr7. (A2) The order reversal of BW27C3 and TAM32-24B23 between r Chr7 and R1 Chr7 clearly demonstrates a large inversion. Staining from a heterozygous R2r bird reveals the same localisations obtained for CH261-95H11 (B1), TAM32-24B23 and BW27C3 (B2) both r Chr7 and R2 Chr7, with CH261-5G3 showing an additional localisation on R2 Chr7 (B1), consistent with a translocated duplication of a segment from the 23.88 MB region to the 16.50 MB region. (TIF) [file pgen.1002775.s003.tif]

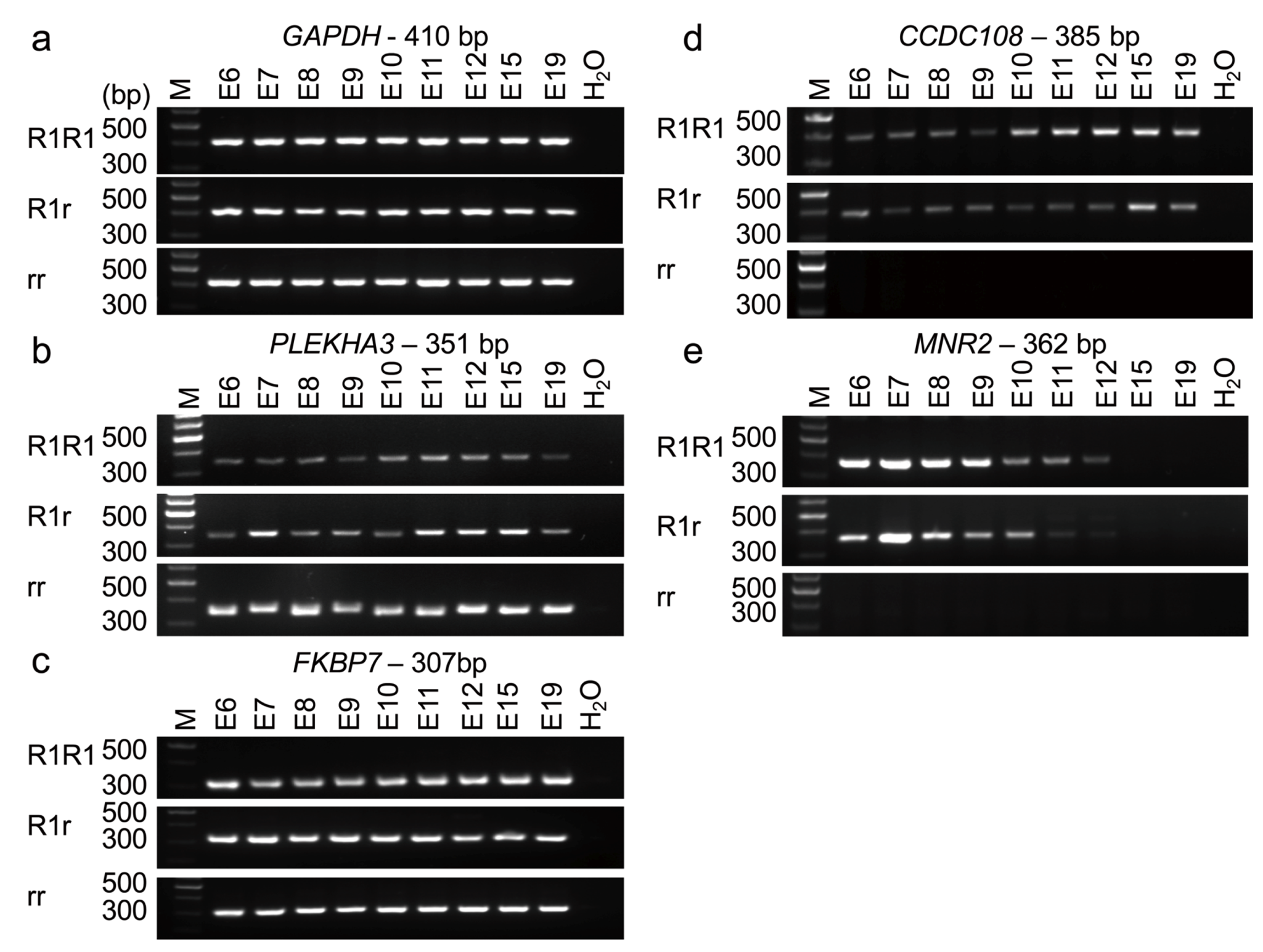

Supplement: Figure S4 — RT-PCR analysis of CCDC108, GAPDH, FKBP7, MNR2 and PLEKHA3 using embryonic comb tissue from single-combed wild-type (rr) and Rose-combed (R1r and R1R1) chickens. E6–E19 represent embryonic days 6 to 19. M = molecular weight marker. H2O = negative control. (TIF) [file pgen.1002775.s004.tif]

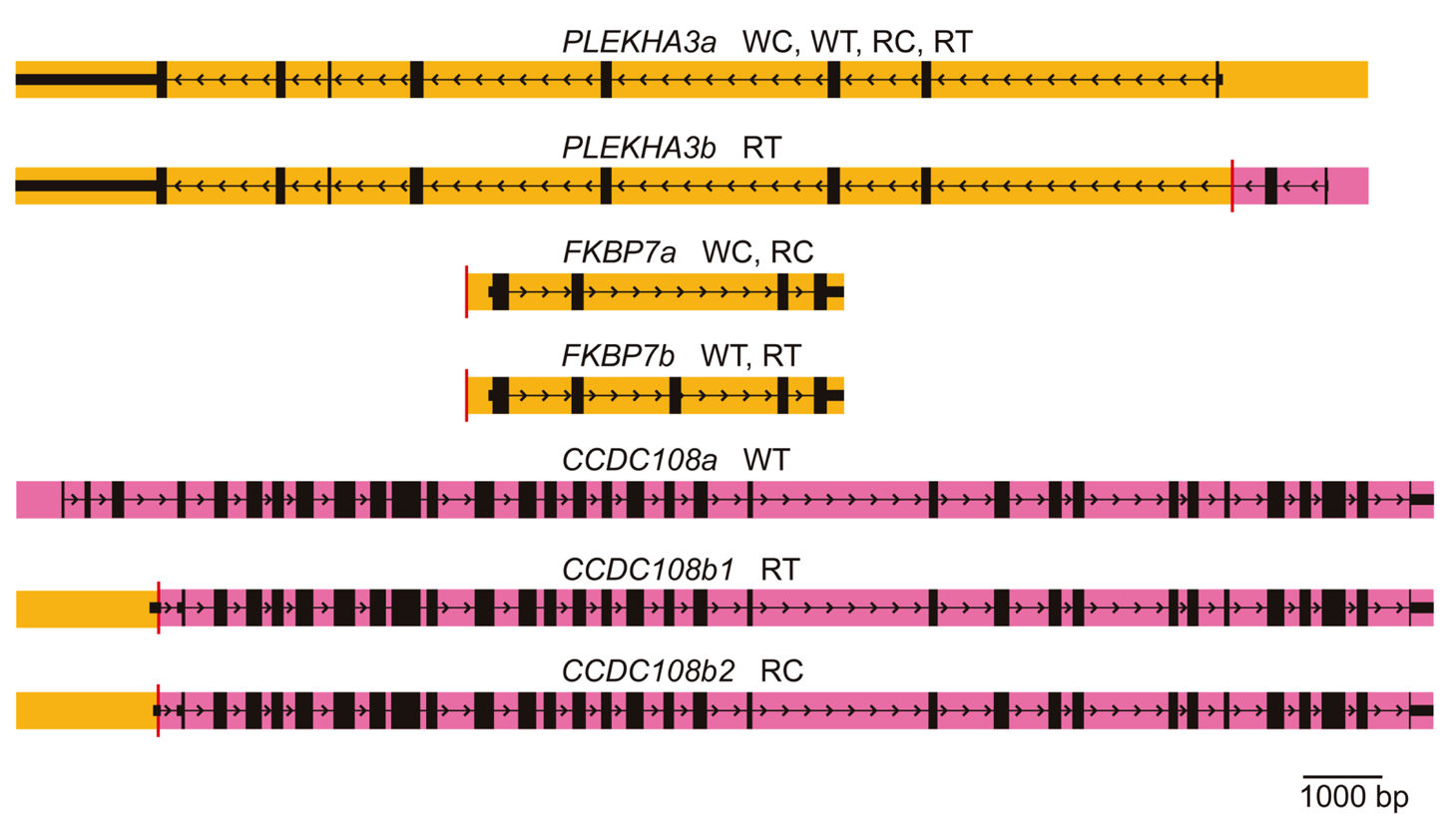

Supplement: Figure S5 — Schematic presentation of the 5′RACE products obtained using embryonic comb tissue and adult testis from single-combed wild-type and Rose-combed (R1R1) homozygotes. Three genes (PLEKHA3, FKBP7 and CCDC108) located in the vicinity of the two R1 inversion breakpoints were investigated. Red vertical bars represent inversion breakpoints. The chromosomal background colour code is consistent with the one used in Figure 3, yellow represents sequences from the 16.50 Mb region and pink sequences from the 23.88 Mb side. WC = wild-type comb tissue; WT = wild-type testis; RC = Rose-comb tissue; RT = Rose-comb testis. (TIF) [file pgen.1002775.s005.tif]

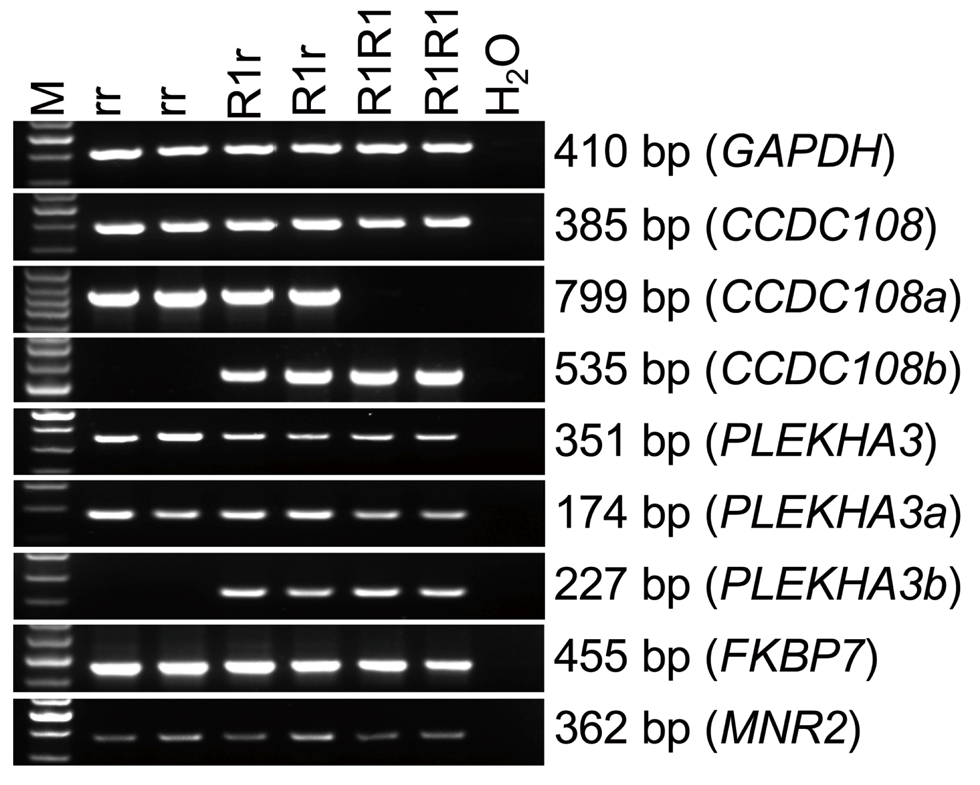

Supplement: Figure S6 — RT-PCR analysis of different transcripts in testis. Homozygous single-combed wild-type (rr), heterozygous (R1r) and homozygous (R1R1) rose-combed animals were used. GAPDH was used as a positive control. The CCDC108 amplicons include exons 15–17, present in both wild-type and mutant transcripts (see Figure S5). The PLEKHA3 amplicons include exons 4–7, present in both PLEKHA3a and PLEKHA3b. Full length FKBP7 and MNR2 transcripts were detected in all three genotypes. (TIF) [file pgen.1002775.s006.tif]

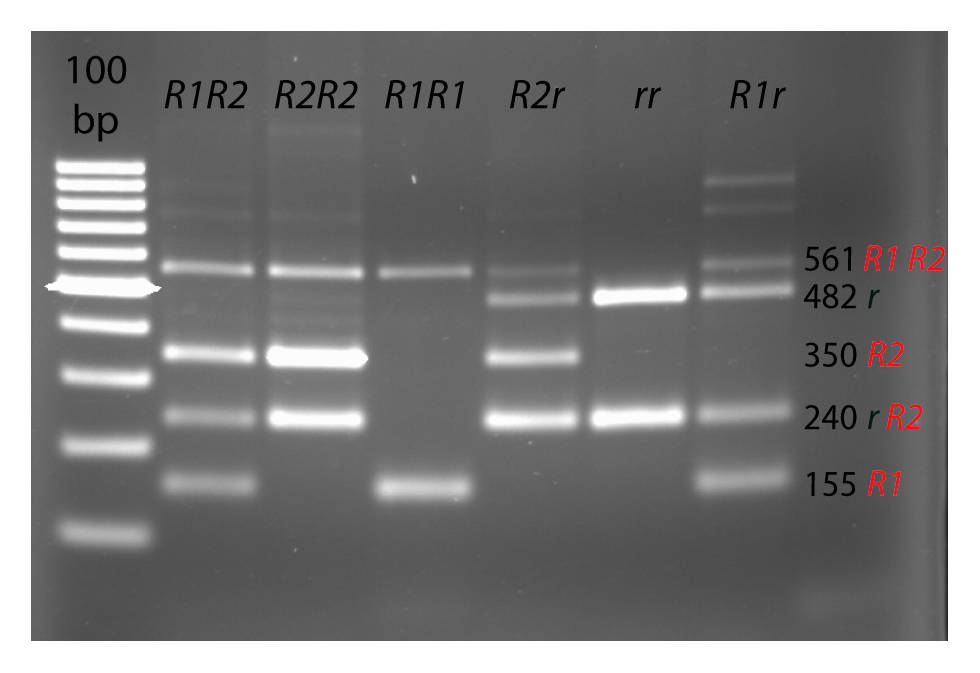

Supplement: Figure S7 — Gel images of electrophoresed PCR products, showing the results obtained with the diagnostic test detailed in Table S4, for the six different genotypes at the Rose-comb locus in chicken. Fragment sizes (in bp) as well as their association to different alleles are indicated to the right. (TIF) [file pgen.1002775.s007.tif]
