# Supplementary material for: The Rose-comb Mutation in Chickens Constitutes a Structural Rearrangement Causing Both Altered Comb Morphology and Defective Sperm Motility
Source: PLoS Genet. 2012 Jun 28;8(6):e1002775. doi: 10.1371/journal.pgen.1002775 (PMC3386170; doi:10.1371/journal.pgen.1002775)
Supplement: Table S1 — Fertility data for different matings involving Rose-comb chicken. (PDF) [file pgen.1002775.s009.pdf]

Table S1 Fertility data for different matings involving Rose-comb chicken.

	Males	Females	# fertile eggs	# infertile eggs	Fertility rate
A	Barbezieux *rr* males (n=15)	Gauloise Grise *rr* females (n=30)	115	67	63.2%
		Charollaise *R1*- females (n=30)	87	87	50.0%
		**Total (n=60)**	**202**	**154**	**56.7%**
	Charollaise *R1R1* males (n=7)	Gauloise Grise *rr* females (n=14)	43	37	53.8%
		Charollaise *R1*- females (n=14)	19	60	24.1%
		**Total (n=28)**	**62**	**97**	**39.0%**
	Charollaise *R1r* males (n=7)	Gauloise Grise *rr* females (n=14)	52	26	66.7%
		Charollaise *R1*- females (n=14)	39	29	57.4%
		**Total (n=28)**	**91**	**55**	**62.3%**
B	Rhode Island Red *rr* males (n=2)	White Leghorn *rr* females (n=10)	**160**	**15**	**91.4%**
	Alsacienne *R2R2* males (n=2)	White Leghorn *rr* females (n=8)	**141**	**19**	**88.1%**
