# Supplementary material for: The Rose-comb Mutation in Chickens Constitutes a Structural Rearrangement Causing Both Altered Comb Morphology and Defective Sperm Motility
Source: PLoS Genet. 2012 Jun 28;8(6):e1002775. doi: 10.1371/journal.pgen.1002775 (PMC3386170; doi:10.1371/journal.pgen.1002775)
Supplement: Table S2 — Compilation of primer sequences used for the Rose-comb locus. (PDF) [file pgen.1002775.s010.pdf]

Table S2 Compilation of primer sequences used for the *Rose-comb* locus.

A. Primers used for microsatellite analysis

Marker	Forward primer	Reverse primer	Forward primer with M13 tag
MCW0120	5'-CTATGTAAAGCTTGAATCTTCA-3'	5'-ATTCCTGGGTGCTAATTTACC-3'	5'-CACGACGTTGTAAAACGACCTATGTAAAGCTTGAATCTTCA-3'
ADL0107	5'-ATTATCCATCCACTTGAGAA-3'	5'-TATTTTTTGAACATTACCAG-3'	5'-CACGACGTTGTAAAACGACATTATCCATCCACTTGAGAA-3'

B. Primers used for SNP analysis using a TaqMan Genotyping Assay

Assay	Forward Primer	Reverse Primer	Reporter 1 Dye	Reporter 1 Sequence	Reporter 1 Quencher	Reporter 2 Dye	Reporter 2 Sequence	Reporter 2 Quencher
C7C19.217	5'-CCCAACTTAGAGCATCACAGAAAGA-3'	5'-GTGATCGAGCACTGGAAAACAC-3'	VIC	5'-AAAGAAAGTGAGCTTGTC-3'	NFQ	FAM	5'-AAAGAAAGTGAACTTGTC-3'	NFQ
C7C19.249	5'-CATTCCCTAGCAAGGAGAAGCT-3'	5'-TCATAGCCTGTCCCCACGAT-3'	VIC	5'-CAAATTGAGCGTTTGTAAC-3'	NFQ	FAM	5'-CAAATTGAGCATTTGTAAC-3'	NFQ
C7C19.253	5'-TTCAGACAAAAATCAAGTCCTCAGAGAT-3'	5'-CAGTTAAAATGGTGGACAGATTTGGT-3'	VIC	5'-AACAAGCTAAGAGGTTC-3'	NFQ	FAM	5'-ACAAGCTAAAAGGTTC-3'	NFQ
KIAA1715	5'-GCTGGTATCAAAAGTCTATACAACTATGAACT-3'	5'-ACTCCTTAAGGCCAACAAAATCTGT-3'	VIC	5'-TTTTAAAGTTGGTTTATAAACAG-3'	NFQ	FAM	5'-AAGTTGGTTTGTAAACAG-3'	NFQ
PTD004	5'-GGAGGTAATTTTGCCATAATTTTTCTGAAGAAC-3'	5'-CGCTTTTGGTCTCATGCAATTTTCA-3'	VIC	5'-TCATGAAAGCGTAAGTGT-3'	NFQ	FAM	5'-TCATGAAAGCATAAGTGT-3'	NFQ
C7C19.442	5'-AAGCCCAAAACTGGACATAGTACTC-3'	5'-TGTGTGAGGGAGGTCATCCT-3'	VIC	5'-CTGCCCCGGTGAGAT-3'	NFQ	FAM	5'-CTGCCCCAGTGAGAT-3'	NFQ
C7C19.559	5'-AGCACCTTCCACTGCTTCAT-3'	5'-CAGAGCAATGCAATAATCCAAGAATCTAA-3'	VIC	5'-CTGAGACACTGGAGTAT-3'	NFQ	FAM	5'-TGAGACACTGGAATAT-3'	NFQ
C7C107.36	5'-ATGTATTATGCAACTCAATGTGGAGGAA-3'	5'-CAGCAGATATATTTGGACTTAGCACACT-3'	VIC	5'-ATGGGCCATCAAAT-3'	NFQ	FAM	5'-CATGGGCCTTCAAAT-3'	NFQ
C7C15.222	5'-TGTTTTCCTGTTGCCCTGAATGT-3'	5'-CCAGAAGGCTAAAACGTCATCTACT-3'	VIC	5'-CGGAGCCTGGGACT-3'	NFQ	FAM	5'-CCGGAGCCTAGGACT-3'	NFQ

C. Primers used for expression analysis

Primer name	Primer sequence
GAPDH-1F	CCCCCAATGTCTCTGTTGTT
GAPDH-1R	GGAACAGAACTGGCCTCTCA
CCDC108-ex15F	GCATGGACTTTTACCCCTGA
CCDC108-ex17R	GCCTGACTGTTACTCGCACA
CCDC108-ex1aF	AACGGGAGTGATGCTACGTG
CCDC108-ex1bF	AAGGGGCAGATGTCCTTCC
CCDC108-ex7R	CTGTGCACCTTTGTGCTGTT

MNR2-ex1F	GCTGGAGAACCAGTTCAAGC
MNR2-ex2R	CATCGTCCTCCTCTTCCTCA
FKBP7-ex1F	AAGGGGGACCTGCTGAAC
FKBP7-ex3R	ATGCTTCAACACTGCGAGGT
PLEKHA3-ex4F	TTCACCACGATGAGACTTGC
PLEKHA3-ex7R	CCGGAGGAGTATCGCTGTAA
PLEKHA3-ex1aF	GGAAGGAGTCCTGTACAAGTGG
PLEKHA3-ex3R	GTGTTGTCTGTTGCATGGACTT
PLEKHA3-ex2bF	ATCCTCGAGAAAGGCACAGA
PLEKHA3-ex2R	GCCTTTGCAAACATCATCCT

D. Primers used for RACE analysis

Primer name	Primer sequence
PLEKHA-5RACE-1R	ATGTTCTCAATGCTGGGAGGAGAGC
PLEKHA-5RACE-2R	CTGCTAAACAGGCCTTTGCACTTC
FKBP7-5RACE-1R	TTTGGTGGAATCTTGCCCTGTGC
FKBP7-5RACE-2R	TCCGTTTTTCTCCAGGACAC
CCDC108-5RACE-1R	ACACGTTGCTATCACGTTGCACACC
CCDC108-5RACE-2R	GTGCTCAGGAGTCATGGAGAAGG
PLEKHA3-3RACE-1F	GTGCCTACTAGCACCTTGATCCAT
PLEKHA3-3RACE-2F	CAGTGCTGTCACTTGTGCTCAGTTC
FKBP7-3RACE-1F	GCAGTAAATAAGGGACCTCGCAGTG
FKBP7-3RACE-2F	AGAAGAACGACCACGATGGAGATG
CCDC108-3RACE-1F	GCAGATAGTGACGGACCTGCTGATA
CCDC108-3RACE-2F	AGCAAAGACCCAACACAGAGGACAG
