# Supplementary material for: The Rose-comb Mutation in Chickens Constitutes a Structural Rearrangement Causing Both Altered Comb Morphology and Defective Sperm Motility
Source: PLoS Genet. 2012 Jun 28;8(6):e1002775. doi: 10.1371/journal.pgen.1002775 (PMC3386170; doi:10.1371/journal.pgen.1002775)
Supplement: Table S3 — Genomic coordinates of BACs used for FISH imaging. BAC BW27C3 has been screened for a genetic marker (MCW201) in the indicated location, but the precise extent of the BAC is unknown, as the ends have not been sequenced. (PDF) [file pgen.1002775.s011.pdf]

Table S3 Genomic coordinates of BACs used for FISH imaging. BAC BW27C3 has been screened for a genetic marker (MCW201) in the indicated location, but the precise extent of the BAC is unknown, as the ends have not been sequenced.

BAC ID	galGal3 Location
CH261-95H11	chr7:16391149-16620754
TAM32-24B23	chr7:16860577-17007780
BW27C3	chr7:22476221-22476531
CH261-5G3	chr7:23742304-23936571
