# Supplementary material for: The Rose-comb Mutation in Chickens Constitutes a Structural Rearrangement Causing Both Altered Comb Morphology and Defective Sperm Motility
Source: PLoS Genet. 2012 Jun 28;8(6):e1002775. doi: 10.1371/journal.pgen.1002775 (PMC3386170; doi:10.1371/journal.pgen.1002775)
Supplement: Table S4 — Diagnostic PCR-based screening of breakpoints associated with the Rose-comb alleles. (PDF) [file pgen.1002775.s012.pdf]

Table S4 Diagnostic PCR-based screening of breakpoints associated with the *Rose-comb* alleles.

A. Primer sequences for breakpoint analysis PCR

Primer	Sequence	Strand in WT
Rose-16.5-out	CAGAGCCCCCAGCGAA	+
Rose-16.5-in	GAAGTGAAACGGCCGTGAG	-
Rose-23.79	GCTGTTGATGCTGCTGGTG	-
Rose-23.88-in	CTGTCTGTGAGGCATTTCCAGT	+
Rose-23.88-out	CTAAATACCTGCCCCTTCCTGA	-

B. PCR mix for breakpoint analysis

Reagent	Volume (µl)
5X Kapa2G GC Buffer w/ 1.5mM $MgCl_2$	2
dNTPs (20 mM)	0.1
Rose-16-out (10 µM)	0.2
Rose-16-in (10 µM)	0.3
Rose-23.79 (10 µM)	0.1
Rose-23-in (10 µM)	0.4
Rose-23-out (10 µM)	0.4
Kapa2G Robust HotStart DNA Polymerase (5U/µl)	0.08
DNA (10 ng/µl)	1
dd H_2O	5.42
Total	10

C. PCR cycle conditions for breakpoint analysis

	Temp	Time
	95°C	5:00
	95°C	0:30

26x	68°C	0:30	-0.5°C/cycle
	72°C	1:00	
	95°C	0:30	
9x	55°C	0:30	
	72°C	1:00	
	72°C	7:00	
	8°C	∞	

D. Result breakdown for breakpoint analysis

Genotype:	*R1R2*	*R1R1*	*R2R2*	*rr*	*R2r*	*R1r*		Primer1	Primer2
	155	155				155	23.89 *R1* inversion band	Rose-23-out	Rose-16-in
	240		240	240	240	240	23.89 *r/R2* band	Rose-23-in	Rose-23-out
	350		350		350		23.79 *R2* duplication band	Rose-23.79	Rose-16-in
				482	482	482	16.5 *r* band	Rose-16-out	Rose-16-in
	561	561	561		561	561	16.5 *R1/R2* inversion band	Rose-16-out	Rose-23-in
