# Supplementary material for: The Rose-comb Mutation in Chickens Constitutes a Structural Rearrangement Causing Both Altered Comb Morphology and Defective Sperm Motility
Source: PLoS Genet. 2012 Jun 28;8(6):e1002775. doi: 10.1371/journal.pgen.1002775 (PMC3386170; doi:10.1371/journal.pgen.1002775)
Supplement: Text S1 — High-resolution mapping of the Rose-comb locus using a second pedigree. (PDF) [file pgen.1002775.s013.pdf]

Text S1 High-resolution mapping of the *Rose-comb* locus using a second pedigree. A White Plymouth Rock x Chinese Silkie intercross comprising 229 F_2 chickens established at China Agricultural University (CAU) was used to map the *Rose-comb* locus segregating in this pedigree. Linkage mapping using a set of 125 microsatellites covering 24 chromosomes revealed highly significant LOD scores to markers on chromosome 7. The entire pedigree was then genotyped with an Illumina 60K SNP array. A linkage map of chromosome 7 (38.4 Mb), comprising 1,882 informative SNPs, was generated. The total map length for chromosome 7 was estimated at 104 cM, which is in good agreement with the estimated 113cM in the current consensus map using a similar set of markers[1]. The linkage analysis provided strong evidence for suppression of recombination in *Rose-comb* heterozygotes as no recombination event was detected in the interval between the markers Gga_rs16586453 at position 14,702,223 bp and GGaluGA315734 at position 23,996,848 bp. The maximum LOD score obtained at 0% recombination was 45.5 for all markers that were fully informative and with no missing genotyping calls.

References

1. Groenen, M. et al. A high-density SNP based linkage map of the chicken genome reveals sequence features correlated with recombination rate. *Genome Res.* **19**, 510-519 (2009).
