# Supplementary material for: The Rose-comb Mutation in Chickens Constitutes a Structural Rearrangement Causing Both Altered Comb Morphology and Defective Sperm Motility
Source: PLoS Genet. 2012 Jun 28;8(6):e1002775. doi: 10.1371/journal.pgen.1002775 (PMC3386170; doi:10.1371/journal.pgen.1002775)
Supplement: Text S2 — Test matings to assess male fertility. (PDF) [file pgen.1002775.s014.pdf]

Text S2 Test matings to assess male fertility. Fifteen single-combed French Barbezieux males (*rr*), seven homozygous Rose-combed Charollaise males (*R1R1*) and seven heterozygous Rose-combed Charollaise males (*R1r*) were each mated to two single-combed Gauloise Grise females (*rr*) and two Rose-combed Charollaise females of unknown zygosity (*R1-)*. Eggs were collected for 10 days after a single insemination of each female. Fertility of eggs was determined by candling at 8 days. Clear eggs were broken to distinguish early embryonic mortality from unfertilised eggs. Early dead embryos were counted in the 'fertile' group. Fertility rate differed significantly between the 3 groups ($\chi2=19.5$ for 2 df, $P<0.001$). Fertility of heterozygous *R1* males was the highest. Single-combed Barbezieux males exhibited a lower fertility rate than what is normally observed for single-combed roosters (Supplementary Table 1). Within the Charollaise breed, fertility rate of homozygous *R1R1* roosters was significantly lower than that of heterozygous *R1r* roosters ($\chi2=16.5$ for 1 df, $p<0.001$).The Charollaise dams exhibited a lower fertility rate than the Gauloise Grise dams, which could be a breed effect, this was significant only when they were mated to Barbezieux males ($\chi2=6.28$ for 1 df, $p<0.02$) or to homozygous *R1R1* Charollaise males ($\chi2=14.7$ for 1 df, $p<0.001$). There was no significant effect of the dam genotype when they were mated to heterozygous Rose-combed Charollaise males, which exhibited the best fertility.

Two single-combed Rhode Island Red males (*rr*) and two homozygous Rose-combed Alsacienne males (*R2R2*) were each mated to five and four single-combed White Leghorn females (*rr*), respectively. Females were inseminated twice a week, eggs were collected in three batches of one week each and fertility determined as described above. In case of a double-yolked egg, each yolk was counted as an observation (fertile, dead or unfertile). No statistical difference was found for fertility between *R2R2* Rose-combed Alsacienne roosters and *rr* single-combed Rhode Island Red roosters.
